# Evaluating Mobile Apps and Biosensing Devices to Monitor Physical Activity and Respiratory Function in Smokers With and Without Respiratory Symptoms or Chronic Obstructive Pulmonary Disease: Protocol for a Proof-of-Concept, Open-Label, Feasibility Study

**DOI:** 10.2196/16461

**Published:** 2020-03-26

**Authors:** Almaz Sharman, Baurzhan Zhussupov, Dana Sharman, Irina Kim

**Affiliations:** 1 Kazakhstan Academy of Preventive Medicine Almaty Kazakhstan; 2 Synergy Research Group Kazakhstan Almaty Kazakhstan

**Keywords:** COPD, mobile health apps, mHealth, smokers, feasibility study

## Abstract

**Background:**

Chronic obstructive pulmonary disease (COPD) is a global public health problem, and continuous monitoring is essential for both its management as well as the management of other chronic diseases. Telemonitoring using mobile health (mHealth) devices has the potential to promote self-management, improve control, increase quality of life, and prevent hospital admissions.

**Objective:**

This study aims to demonstrate whether a large-scale study assessing the use of mHealth devices to improve the treatment, assessment, compliance, and outcomes of chronic diseases, particularly COPD and cardio-metabolic syndrome, is feasible. This will allow our team to select the appropriate design and characteristics for our large-scale study.

**Methods:**

A total of 3 cohorts, with 9 participants in each, will use mHealth devices for 90 days while undergoing the current standard of care. These groups are: 9 “non-COPD,” otherwise healthy, smokers; 9 “grey zone” smokers (forced expiratory volume in 1 second/ forced vital capacity ≥0.70 after bronchodilator treatment; COPD Assessment Test ≥10); and 9 smokers diagnosed with Stage 1-3 COPD. Rates of recruitment, retention, and adherence will be measured. Overall, two mHealth devices will be utilized in the study: the AnaMed Original Equipment Manufacturer device (measures distance, energy expenditure, heart rate, and heart rate variability) and the Air Next mobile spirometry device. The mHealth devices will be compared against industry standards. Additionally, a questionnaire will be administered to assess the participants’ perceptions of the mHealth technologies used.

**Results:**

The inclusion of participants started in June 2019. Study results will be published in peer-reviewed scientific journals.

**Conclusions:**

This study will demonstrate whether a large-scale study to assess the use of mHealth devices to improve the treatment, assessment, compliance, and outcomes of chronic diseases, particularly COPD and cardio-metabolic syndrome, is feasible. It will also allow the research team to select the appropriate design and characteristics for the large-scale study.

**Trial Registration:**

ClinicalTrials.gov NCT04081961; https://clinicaltrials.gov/ct2/show/NCT04081961

**International Registered Report Identifier (IRRID):**

DERR1-10.2196/16461

## Introduction

Chronic obstructive pulmonary disease (COPD) accounted for 3.2 million deaths globally in 2015 [1] and is the fourth leading cause of death both worldwide and in Kazakhstan [2]. COPD is a heterogeneous condition, with a variety of disease-related phenotypes [3,4], and its main risk factor is cigarette smoking [5]. The chronic airflow limitation that characterizes COPD is caused by obstructive bronchiolitis and parenchymal destruction (emphysema). Pulmonary emphysema is a form of COPD; however, pulmonary emphysema without airway obstruction is common in smokers [6,7]. Smokers with symptoms suggestive of COPD who do not qualify for a diagnosis of COPD based on spirometry are referred to as “grey zone” COPD patients. They have preserved pulmonary function (forced expired volume in 1 second/forced vital capacity [FEV_1_/FVC] of at least 0.70 after bronchodilator and FVC ≥80% of the expected value) and respiratory symptoms (COPD assessment test [CAT] ≥10).

Continuous monitoring is vital for the management of COPD. Implementing telemedicine and mobile health (mHealth) innovations has allowed clinicians to intervene in COPD earlier and prevent complications. However, there remain challenges in the form of alarm frequency and response, both of which need to be implemented into the existing workflow [8]. Data flow and workflow processes need to be designed with precision at the outset if telemedicine is to be applied in clinical practice. Telemonitoring using mHealth devices has the potential to promote self-management, improve control, increase quality of life, and prevent hospital admissions [9-13]. Technological advances in mHealth home telemonitoring (electronic health [eHealth]) programs and systems can affect care for patients with COPD [12,14-17]. mHealth devices are an emerging opportunity in clinical studies, and their utility (ie, sensitivity, accuracy, and reproducibility) has previously been assessed for telemonitoring for COPD [14].

Telemonitoring is a promising alternative or adjunct to the provision of traditional health care services in COPD [18]. Although some studies have shown that telemonitoring may improve some clinical outcomes and reduce health care costs [19,20], the effects of telehealth interventions on emergency department attendance, hospital admissions, duration of admissions, health-related quality of life, costs, and mortality remain less certain [18,21-25].

In a recent study of telemedicine in the home setting using multiple activity sensor monitoring equipment in COPD patients, the augmentation of traditional telemedicine methods with motion sensing, spirometry, and symptom diaries appeared feasible [26]. In a literature review (141 randomized trials; n=37,695) of studies of eHealth practices, such as telemetry, telephone calls, or home visits by nurse specialists, most studies were relatively short term (<6 months) and did not yield strong evidence for telemedicine use in the management of chronic diseases [27]. However, the comparison of outcomes in studies using telehealth applications is difficult due to advances in monitoring and communications technology and heterogeneity in the type of monitoring, the disease entity and severity, and the variations in the process of care brought about by the telemedicine intervention [12].

Although peak flow monitoring has been used for at-home detection of asthma exacerbations, and studies in the past have monitored vital signs and symptoms in patients with COPD [28], few studies have attempted to deploy spirometry for home monitoring of COPD [29]. With technical advances, spirometry is increasingly being used to track the progress of COPD over time and to identify acute exacerbations [30-34].

While the number of COPD mHealth devices is rapidly increasing, most have not been validated as clinically effective tools for the management of the disease. In addition to empowering patients and facilitating disease self-management, mHealth offers promising aid to COPD researchers to help them personalize treatments based on patient-specific profiles and integrate symptom occurrence and medication usage with environmental and genomic data. An integrated and targeted practice-managed approach that uses mHealth technologies in primary care settings will be most effective for the early identification, monitoring, and management of chronic diseases, particularly COPD and cardio-metabolic syndrome (ie, combined diabetes mellitus, systemic arterial hypertension, central obesity, and hyperlipidemia). Health information technologies are revolutionizing health care by assisting patients in self-monitoring and decision-making, driving a shift toward a care model increasingly centered on personal use of digital and web-based tools [35-37]. Because there is a dearth of evidence that direct-to-consumer mHealth tools are effective or that they provide accurate disease recommendations, they are not yet widely used in clinical practice. Nonetheless, the preponderance of mHealth is gradually increasing in health care, industry, and as a subject of research [38].

This study aims to investigate the feasibility and utility of using mHealth devices to improve the treatment, assessment, compliance, and outcomes of smokers with and without respiratory symptoms/COPD. It namely means to assess the feasibility of mHealth devices in current smokers with and without respiratory symptoms or COPD by monitoring physical activity, vital signs, and respiratory function, and aims to assess the validity of mHealth devices in detecting vitality parameters as compared to industry standards.

After demonstrating proof of concept in this study, its purpose will be to incorporate mHealth devices into an ongoing 5-year longitudinal cohort observational study to monitor selected vitality parameters and other comorbidities [39]. Specifically, depending on the outcomes of the study, the AnaMed Original Equipment Manufacturer (OEM) device and the Air Next mobile spirometer will be introduced to record data from a randomized subsample of participants in an observational cohort study, including smokers of combustible cigarettes and users of IQOS with HeatSticks.

## Methods

### Study Design

This is a proof-of-concept, open-label, three-arm, observational, single-center feasibility study. A total of 27 participants in three cohorts will use the mHealth devices for 90 days while undergoing the current standard of care based on their smoking disease state or lack of disease state. The groups are made up of nine “non-COPD,” otherwise healthy, smokers, nine “grey zone” smokers (ie, FEV_1_/FVC ≥0.70 after bronchodilator treatment, CAT ≥10; six-minute walk test [6MWT]<450 meters), and nine smokers diagnosed with Stage 1-3 COPD.

In each group, nine participants will be randomly assigned to three types of reminders: three participants will be reminded every morning by text message or phone call and contacted every evening by phone or chat services (eg, Skype, WhatsApp, Viber, texting) to share their experiences and feedback on mHealth device usage; three participants will receive only morning reminders; and three participants will receive neither morning reminders nor evening communication/feedback.

### Study Devices and Assessments

Two mHealth devices will be utilized in the study: the AnaMed OEM device (measures step counts, energy expenditure, heart rate, and heart rate variability) and the Air Next mobile spirometry device (Nuvoair AB, Stockholm, Sweden) (measures FEV_1_, FVC, and forced expiratory flow).

At the Kazakhstan Academy of Preventive Medicine COPD Center, standard spirometry data are collected by using the BTL-08 SPIRO (BTL Industries Limited, United Kingdom) spirometry system. The spirometer used in this study is tested and continuously standardized with a 3-liter syringe. Quality assessments will be performed throughout the study. The Vive Precision DMD 1003 pulse oximeter is used to get peripheral capillary oxygen saturation (SpO2) and pulse readings at the Kazakhstan Academy of Preventive Medicine COPD center and will be used for comparison to the results produced by the AnaMed OEM device.

### Outcome Measures

Safety and tolerability will be evaluated through adverse events (AEs), lung function tests, vital signs, and supportive care medications. Primary measures are defined as rates of recruitment, retention, and adherence as well as safety of the intervention that are common for feasibility studies [40]. Recruitment is defined as the number of potential participants screened for study eligibility versus the number of people who enrolled in the study. Retention is defined as the proportion of participants enrolled who completed the intervention and all study measures. Adherence to the study protocol is determined as the proportion of participants enrolled who had all their mHealth parameters registered every day.

The mHealth devices will be compared to the industry standards. Additionally, a questionnaire will be administered to assess the participants’ perceptions of the mHealth technologies used.

### Inclusion and Exclusion Criteria

#### Inclusion Criteria

Participants should meet the following criteria to be eligible to enroll in the study:

- 40-59 years of age
- Current smokers who are smoking conventional cigarettes with a minimum of a ten pack-year smoking history (calculated by taking the average number of cigarettes smoked per day divided by 20 and multiplied by the number of years smoked):
  - Asymptomatic current smokers: no symptoms (CAT<10, 6MWT≥450 meters) and preserved pulmonary function based on spirometry (FEV_1_/FVC of at least 0.70 after bronchodilation treatment and FVC ≥80% of the expected value) and respiratory symptoms (CAT ≥10); OR
  - “Grey zone” current smokers: initially preserved pulmonary function based on spirometry, but with clinical symptoms based on CAT (>10) and 6MWT (<450); OR
  - Current smokers with a confirmed diagnosis of COPD (Global Initiative for Chronic Obstructive Lung Disease [GOLD] stage I-III).
- Able to use and willing to be trained to use mHealth devices.
- Provide written, informed consent to participate in the study.

Participants will undergo the current standard of care based on their smoking disease states or lack of disease state.

#### Exclusion Criteria

Participants meeting any of the following exclusion criteria are not eligible to enroll in the study:

- Smokers with COPD exacerbation (defined as a change in symptoms requiring increased doses of current medicines or the prescription of new medicines, such as corticosteroids or antibiotics) that has not resolved at least 28 days before screening. Smokers with COPD exacerbations occurring after screening but before the first study visit should also be excluded.
- Smokers with pneumonia or other respiratory tract infections that have not resolved at least 14 days before screening. Any participant that experiences pneumonia occurring after screening but before the first study visit should also be excluded.
- Smokers with other active respiratory disorders: tuberculosis, lung cancer, significant bronchiectasis, sarcoidosis, bronchial asthma, lung fibrosis, pulmonary hypertension, interstitial lung diseases, or other active pulmonary diseases.
- Any comorbid medical condition that, in the opinion of the investigator, would make participation in the study unsafe or unfeasible. This includes conditions that prohibit completion of exercise testing, such as orthopedic, neurological, cardiovascular, or other conditions that significantly impair standard biomechanical movement patterns and limit the ability to walk/cycle, as judged by the investigator.
- Use of supplemental oxygen therapy.
- Inability to abstain from smoking during the period in which the participant is admitted to the Kazakhstan Academy of Preventive Medicine COPD Center.
- A history of allergies or hypersensitivity to metal, particularly stainless steel.
- Any vital sign indicator, such as hypertension or tachycardia at rest that, at the discretion of the investigator, would make participation in the study unsafe or unfeasible.
- Women who test positive for pregnancy during screening, lactating women, or women planning to become pregnant during the study.
- Participants using assistive devices like walking aids, as these are likely to interfere with physical activity.
- Other patients who are considered ineligible for the study by the investigator.

### Sample Size Calculation

The primary endpoints of the study are rates of recruitment, adherence, and retention. To assess the feasibility of the intervention, we plan to recruit 27 participants, which should be enough to get estimates with a sufficient degree of uncertainty. We conservatively predict that 30% of the people invited to participate will be recruited to the study, with a 95% CI of 13-47%. We also assume the dropout rate will be 15%. The accuracy of the estimated retention rate will be at least ±13%. Further, we believe that 70% of participants will adhere to the use of mHealth devices. In this case, the accuracy of the estimate will be at least 17%. All calculations are based on two-way 95% confidence intervals.

### Study Procedures

#### Overview

The study will last 90 days and has two stages. The first stage includes the initial period of using the mHealth devices (Days 1-21) to evaluate the validity of collecting vitality parameters (eg, heart rate, blood oxygenation, steps/motion) on mHealth devices. The main period of use for the mHealth devices (Days 22-90) is the second stage, which aims to evaluate the feasibility of participants using these devices. The schedule of enrollment and data collection is shown in Table 1.

For Table 1, spirometry was performed to diagnose and monitor COPD. Providing of mHealth devices involved the provision of the AnaMed OEM device, the Air Next mobile spirometer, and instructions/review of how to use these tools (print and verbal instructions). For the assessment of the AnaMed OEM device, the participants’ SpO2 will be measured at each visit using industry-standard pulse oximetry devices, and for the assessment of the Air Next spirometer, participants will host the mobile spirometer at home for once daily measurements. Measurements will be validated at Study Center visits using an industry-standard device before and after the use of a bronchodilator.

**Table 1:** Schedule of study activities.

		Device assessment period			Clinical feasibility study period				
	Screening	Baseline visit	Interim visit	Final visit	Interim visits			Final visit	
Visit	1	2	3	4	5	6	7		8
Days	1	7	14	21	28	35	56		90
Informed consent process	✓								
Study eligibility and smoking status	✓								
Reviewing medical history (including physical examination and BMI^a^ measurement)	✓	✓	✓	✓	✓	✓	✓		✓
COPD^b^ assessment test	✓	✓	✓	✓	✓	✓	✓		✓
Spirometry	✓	✓	✓	✓	✓	✓	✓		✓
6-minute walk test	✓	✓	✓	✓	✓	✓	✓		✓
Providing the study requirements handout and explaining the study/visit requirements	✓	✓	✓	✓	✓	✓	✓		✓
Dichotomous questionnaire for visit readiness	✓								
Providing mHealth^c^ devices	✓								
Assessment of AnaMed OEM^d^ device		Continuous monitoring							
Assessment of Air Next mobile spirometer against standard		Continuous monitoring							

^a^BMI: body mass index.

^b^COPD: chronic obstructive pulmonary disease.

^c^mHealth: mobile health.

^d^OEM: original equipment manufacturer.

#### Participant Recruitment and Registration

We will employ various nonprobability sampling techniques, including quota and snowball sampling methods, to recruit study participants. The Kazakhstan Academy of Preventive Medicine research team will register patients for each mHealth device. Installation and user guides for each technology used include labeled photographs and written instructions to be used by all teams and patients during setup. All equipment has been tested before deployment. Training is provided on setup, installation, and use as well as individual checklists, decision trees, and troubleshooting information. The break for charging is at a standard time (20:00) across arms. In addition to direct phone communication, WhatsApp, texting, and other types of messaging systems are used for sharing daily experiences each evening to assist with assessing the level of comfort and address issues with wearing the AnaMed OEM device and using the Air Next mobile spirometer.

#### Data Collection

Participants will synchronize their wearable device (AnaMed OEM device) and the Air Next mobile spirometry device by signing into their account. Data are stored in a local cloud system. The entire process of data ingestion and storage has been audited, according to ALCOA (attributable, legible, contemporaneously recorded, original, accurate) standards [41]. Whenever a participant synchronizes new activity data to their device cloud, those data would be ingested, processed, and archived, and then aggregated and summarized in JSON data format by summarization services.

#### Mobile Health Apps

Participants will be provided with a smartphone (iPhone) to perform and visualize measurements and are expected to keep smartphones after the study completion, which will serve as compensation for participation. This is reflected in the Informed Consent Form.

Participants will be guided to assess their health status using the Symptomaster application (HealthCity, Kazakhstan) and zdrav.kz database, which will both also act as tools to determine any potential AEs. The SmartHealth technology Symptomaster helps patients to establish the probable causes of the symptoms of diseases without assistance from a healthcare professional. Using a smartphone, a participant inputs his/her symptoms into the system, which then produces the most likely preliminary diagnosis. After receiving the diagnosis, a patient can refer to zdrav.kz, an online library that contains information about the 1000 most common diseases and their causes and symptoms, in addition to ways to prevent and treat them. These technologies allow a participant to make an informed decision about whether they should seek immediate medical assistance by calling an ambulance or if they should consult a doctor on their next routine visit.

#### Physical Examination

Physical examinations will be conducted during each visit based on the Stanford Medicine 25 comprehensive clinical assessment to identify clinical signs of abnormalities. This will be in addition to standard anthropometric measurements and vital sign assessments (pulse rate, blood oxygenation, and blood pressure).

#### Spirometry

The Air Next mobile spirometer will be used by patients to assess respiratory function. To use it, patients must hold their hands on tubular grips or use wrist clamps. Subsequent respiratory efforts allow the determination of inspiratory capacity and FEV_1_. Participants are categorized for analysis using the GOLD staging system according to their spirometry, which will be performed before and after two inhalations of salbutamol (0.1 μg per inhalation). Among the criteria needed to make a diagnosis of COPD are deficits in the rate at which one can forcefully exhale. Most experts consider a low ratio (<0.70) of the FEV_1_ to the FVC after bronchodilator use to be a key diagnostic criterion. Bronchodilator responsiveness will be considered positive if the participant has a ≥12% change in FEV_1_ or FVC above prebronchodilator measurements.

#### Six-Minute Walk Test

This test measures the distance that a patient can quickly walk on a flat, hard surface in 6 minutes. A 100-feet hallway is needed, and no exercise equipment or advanced training for technicians is required.

#### Physical Activity

Study participants will measure their pedometer-determined physical activity using the AnaMed OEM wearable devices. While performing the six-minute walk test, participants will simultaneously use the AnaMed OEM and Garmin Vivo (Garmin Ltd, Olathe, Kansas, United States) devices to compare step counts from both devices.

#### Chronic Obstructive Pulmonary Disease Assessment Test

The CAT is used as an add-on test with existing assessments in COPD (eg, with FEV_1_). It is a simple and reliable measure of health status in COPD as it assists patients and their physicians in quantifying the impact of COPD on the patient’s health. The CAT is a validated, short (8-item) questionnaire to be completed by patients.

#### User Experience Questionnaire

Participants will be administered questionnaires to assess their mHealth device use experience. One questionnaire is administered for each device. The questions will address comfort levels and ease of daily vital measurements. The interviews will be conducted by clinical investigators not involved with the quantitative monitoring or analysis to reduce the possibility of bias.

### Data Management

All study data will be stored in the information technology Unit of the Kazakhstan Academy of Preventive Medicine. Verification of eligibility was completed via a web questionnaire after participants signed the consent form, and participants will be tracked for the completion of all the study data. If a participant is excluded or discontinues use during or after the study procedures, the specific exclusion or discontinuation reason will be recorded in the database.

All electronic files are encoded using a 128-bit advanced encryption standard and are password protected on a computer with both hardware and software firewalls. The locator form and any documents with identifying information are kept in a separate folder and kept locked in filing cabinets.

### Statistical Analysis

For this proof-of-concept phase, access to device-derived data will be enabled via a cloud-to-cloud solution. Graphical and statistical comparisons will be made between the mobile biosensing device–derived data and the data derived from the clinical standards, and between the three different study groups. Descriptive statistics will be used to summarize required qualitative and quantitative study elements (eg, proportion, mean, standard error, median, interquartile range, 95% confidence interval).

The exploratory graphical analysis will be done before the numerical analysis. Histograms and two-dimensional scatterplots of raw data will provide information on the univariate and bivariate distributions of the variables, focusing on the distribution of variables and relationships between the variables (whether there is a linear or nonlinear relationship). Additionally, preliminary graphs will be used to screen raw data by highlighting obvious data errors. Tabulations will be produced for appropriate disposition, demographics, baseline, safety, and clinical parameters.

Statistical comparisons will be made between the mobile biosensing device–derived data and the data derived from the standard diagnostic equipment and methods. Agreement analysis will be performed for both binary and quantitative measures. For binary variables, percent of agreement (overall, positive, and negative agreement) as well as Kappa coefficient, *P* value, and 95% confidence interval will be calculated. For two quantitative measures of a parameter, we will use the Bland-Altman method (Bland-Altman plot and limits of agreement). The Bland-Altman plot analysis will allow us to evaluate a bias between the mean differences and to estimate an agreement interval, within which 95% of the differences between two quantitative methods of measurement are included. Correlation analysis will also be run so that Pearson’s correlation coefficient and the 95% confidence interval will be calculated.

The agreement analysis will be done for baseline, 7-day, 14-day, 21-day, 28-day, 56-day, and 90-day visits separately and for the data pooled from all measurements. Within-Subject study design will be accounted for to assess accuracy and precision for a single mobile device. All statistical analyses will be done for all participants and by study group. Additionally, we will compare trends of binary and quantitative outcomes from three study groups wearing mobile devices.

The analysis will be performed using R Statistical Software (R Foundation for Statistical Computing, Vienna, Austria).

### Ethics Approval

The Ethics Committee of the Academy of Preventive Medicine approved this study on June 3, 2019. The study has been registered at ClinicalTrials.gov (NCT04081961).

## Results

The inclusion of the participants started in June 2019. Study results will be published in peer-reviewed scientific journals.

## Discussion

### Principal Considerations

The proposed study is the first step in a series of studies aiming to investigate the effect of using mHealth devices to improve the treatment, assessment, compliance, and outcomes of smokers with and without respiratory symptoms/COPD. The results from this proof-of-concept, open-label, device feasibility study will be used to finalize the protocol for a randomized, open-label, placebo-controlled, single-center, two-arm, 12-month study designed to assess clinical feasibility and the effect of this intervention.

### Limitations

This study is a small-scale, exploratory, pilot study which is looking to answer questions about whether a larger trial is feasible or not and seeks to get estimates of parameters required for the calculation of the sample size of the main study. The results of this study cannot be used to estimate the effect size of using mHealth devices because the sample size is too small.

### Conclusion

Many studies have shown that mHealth tools are effective or that they provide accurate disease recommendations. This study will demonstrate whether a large-scale study to assess the use of mHealth devices to improve the treatment, assessment, compliance, and outcomes of chronic diseases, particularly COPD and cardio-metabolic syndrome, is feasible, and will also allow for the selection of an appropriate design and characteristics for the later large-scale study.

## Abbreviations

6MWT: six-minute walk test
AE: adverse event
CAT: Chronic Obstructive Pulmonary Disease Assessment Test
COPD: chronic obstructive pulmonary disease
eHealth: electronic health
FEV_1_: forced expiratory volume in 1 second
FVC: forced vital capacity
GOLD: Global Initiative for Chronic Obstructive Lung Disease
mHealth: mobile health
OEM: original equipment manufacturer
SpO2: peripheral capillary oxygen saturation
